# Supplementary material for: Arresting visuospatial stimulation is insufficient to disrupt analogue traumatic intrusions
Source: PLoS One. 2020 Feb 3;15(2):e0228416. doi: 10.1371/journal.pone.0228416 (PMC6999047; doi:10.1371/journal.pone.0228416)
Supplement: Attachment — Submitted filename: ResponseToReviewers_R1 11_2019.docx [file pone.0228416.s001.docx]

Ilan Harpaz-Rotem, PhD

Academic Editor

PLOS ONE

Date: 20.11.2019

Response to reviewers

**Journal Requirements:**

1) Please ensure that your manuscript meets PLOS ONE's style requirements, including those for file naming.

**Response:** We checked style requirements and also updated the file names accordingly.

2) Please amend your current ethics statement to confirm that your named ethics committee Institutional Care and Use Committee (IACUC) specifically approved this study. Once you have amended this/these statement(s) in the Methods section of the manuscript, please add the same text to the “Ethics Statement” field of the submission form (via “Edit Submission”).

**Response:** The ethics statement has been updated to clarify that: “All participants gave written informed consent prior to inclusion. Study 1 and Study 2 were approved by the research ethics committee of the Behavioural Science Institute, Radboud University Nijmegen (ECSW2015-1105-310).”

**Editor Comments**

1) The manuscript is interesting and carries important findings that need to be further investigated and interpreted correctly. Both reviewers raised several significant concerns that need to be addressed by the authors. Please respond carefully to all the reviewers comments in the revised manuscript.

**Response:** We would like to thank the editor and the reviewers for their time and effort and consideration of our manuscript. We carefully considered the comments and suggestions that have been raised and revised the paper accordingly to address each point.

**Reviewer #1**

1) Intrusive memories are a hallmark feature of post-traumatic stress disorder (PTSD), however, the frequency of intrusive memories can be reduced if particular behavioral tasks (e.g., the computer game Tetris) are administered following a traumatic event. Importantly, not all behavioral tasks reduce intrusive memories following traumatic experiences and the mechanisms driving the effect are currently unknown. Thus, work that outlines the boundary conditions that define successful behavioral manipulations is critical. The paper under review seeks to identify such parameters. To investigate whether the visuospatial elements of behavioral tasks are the driving force behind tasks that successfully reduce intrusive memories, this paper tested the hypothesis that viewing attention-grabbing visuospatial stimuli during consolidation of a trauma memory reduces the frequency of intrusive memories. Intrusive memories were induced by an analogue trauma film within a healthy population and were measured using a self-report diary in which participants tracked post-film intrusive memories for one week. The paper reports null findings – the frequency of intrusive memories was the same for control and experimental conditions and the paper concludes that viewing visuospatial stimuli is not sufficient to disrupt intrusive memories. The paper additionally explored whether reduced frequency of intrusive memories is dependent on an interaction between the visuospatial stimulation task and 1) working memory capacity, 2) working memory load during the visuospatial task, and 3) concurrent trauma memory reactivation. It found no interaction effects – the frequency of intrusive memories was the same across all conditions.

**Response:** Thank you for this elaborate and accurate summary, as well as for sharing our appreciation of research into the boundary conditions of behavioural interventions against intrusive memories.

2) However, since the employed protocol did not mimic the same types of visuospatial stimuli known to reduce intrusions, it is difficult to draw firm conclusions from the work. For example, it may be the case that passively viewing a pre-recorded Tetris game successfully reduces intrusive memories, even though the stimuli used in the present study were not able to do so. Additionally, stimuli from Tetris are often mentally replayed even after the game is complete. After the game, players report seeing the world as a game of Tetris (e.g., visualizing falling shapes or imagining how objects in their environment could be rotated to fit together). One formal study noted intrusive, stereotypical, visual images during sleep after Tetris play. This was also the case within amnesic patients when there was no declarative memory of real-life gameplay (Stickgold et al., 2000, Science). Thus, it may be the case that visuospatial stimuli are able to disrupt intrusive memories, but only when the visuospatial stimuli are replayed in the days following the trauma film. Mental replay that extends over days may have a more potent impact on consolidation processes or more aggressively compete for resources needed for consolidation. It would be helpful to know if mental images of the visuospatial stimuli used in the present study induced replay during wakefulness and/or sleep, and if so, how the frequency of such intrusions compare to that induced by passive Tetris-watching and active Tetris gameplay.

**Response:** We thank the reviewer for these thoughtful considerations and agree that mental replay could be a highly relevant factor in the context of Tetris and intrusions. Unfortunately, we do not know whether the visuospatial stimuli we used induced any form of mental replay, since we did not include a task/measure of spontaneous imagery involving these stimuli. Given this important comment, however, we have extended the section addressing directions for future research accordingly (pp.27-31).

In particular, we now discuss that factors such as spatial planning, updating, or coordination of motor movements could be controlled for more directly in future studies, “*e.g., by comparing actual Tetris gameplay to passive Tetris-watching*” (lines 640-642).

Inspired by the reviewer’s comment, we also include the interesting idea that interventions like Tetris could have effects after rather than during gameplay: “… *high levels of flow experience may result in altered experiences even after gameplay, including continued replay, as well as intrusive visual images of the game [58]. Speculatively, the beneficial effects that have been reported for Tetris may thus depend on competition for visuospatial resources after, rather than during gameplay. Therefore, an exciting avenue for future studies would be to explore if a task’s ability to induce a flow experience, continued replay, and/or intrusive imagery is required in order to interfere with intrusive memories of a trauma film*” (lines 648-655).

3) As a separate comment, several of the studies cited in this paper are of manipulations employed during a memory’s reconsolidation, not consolidation, phase. Often times the paper does not clarify when findings from reconsolidation studies are being used to justify the rationale of this consolidation study. Additionally, the cited review articles are skewed toward manipulations that occur during trauma movie watching, rather than during the consolidation period. Since there appear to be fewer studies that have employed manipulations during the consolidation period, it may be helpful to prioritize discussing those specific experiments over referencing less-relevant work. It would specifically be useful to discuss behavioral manipulations employed during the consolidation period that did not lead to reductions in intrusion frequency. Currently, this is not thoroughly discussed, but if included, could emphasize the nuance needed when studying manipulations as such and clarify why the null findings of the present study are not completely at odds with the currently available literature.

**Response:** We agree that a more specific focus on studies targeting the consolidation phase following trauma film viewing is useful and have revised the introduction and discussion sections accordingly. In particular, we now discuss a series of studies that employed the game Tetris “*immediately after a viewing a trauma film [7], after 30 min [e.g., 8, 9], or after 4 h [10]*” (lines 51-53), and elaborate on their common rationale that “*newly formed memories are prone to retroactive interference for up to several hours [11, 12]*” (lines 56-57). In addition, we now emphasize more clearly that our paper addresses interventions during the consolidation phase following trauma film viewing throughout the entire manuscript and draw a more explicit distinction with re-consolidation/re-encoding studies (e.g., lines 117-118; 372-376; 526-527).

Furthermore, we have included two relevant recent papers (Asselbergs et al., 2018, EJPT; van Schie et al., 2018, BRAT) that employed different visuospatial tasks during the consolidation period following a trauma film. These papers reported null findings across a series of studies and indeed help to clarify that the null finding of our own experiments are not entirely at odds with the available literature. As we now elaborate both in the introduction (lines 82-99) and the discussion (lines 584-600), the studies reported in both papers suggest that the effectiveness of visuospatial tasks to reduce intrusive memories cannot be entirely attributed to working memory taxation and/or individual differences in working memory capacity. Next to our own work, these studies further emphasize the need for additional research that we discuss in detail in the section “Future directions”.

**Reviewer #2**

1) In this carefully-conducted piece, the authors attempt to replicate and extend findings from a growing literature indicating that intervening with a perceptual task after trauma-film viewing decreases numbers of intrusive thoughts compared to no-task conditions. The authors attempt to manipulate the likely components necessary to produce this effect: 1) distraction; and 2) working memory load. Despite a well-conducted and adequately-powered study, the authors fail to replicate past findings.

**Response:** Thank you for this summary and for the encouraging words concerning our methodology.

2) Overall, I think this is an important contribution to the literature that could be made even more impactful with the inclusion of one more study, as below:

The authors demonstrate that intervention by neither working memory load manipulation (controlled also for baseline working memory capacity) nor distraction is capable of producing decreased frequency of intrusive memories after trauma film viewing. The authors do an admirable job of discussing various components that could be important for replication of these findings, but do not consider what may be the most obvious: the need for both visually-arresting and working-memory-intensive tasks.

Without this control, the results of the study are interesting and worth reporting; however, with it, the authors say something truly impactful about the mechanisms necessary to achieve a decrease in intrusive memories. The authors should conduct this experiment, or explain why they think the current results are able to speak to the possibility that an interaction between these two components is necessary to achieve the desired results.

**Response:** We fully agree that based on Study 1, one could expect visually arresting stimulation to be effective only if it is combined with intensive working memory load. Indeed, the design of Study 2 was based on this reasoning: in both active conditions, participants were exposed to the visually arresting stimuli whilst executing the WM-intensive tasks involving memorizing and imagining.

Strictly speaking, it is correct that Study 2 did not formally test the interaction between high/low WM load on the one hand, and high/low visually arresting stimuli on the other hand. However, Study 1 tested low WM load combined with H-VA vs. L-VA stimuli, while Study 2 addressed high WM load combined with H-VA stimuli. Importantly, both studies included no-task control groups, and none of the active interventions differed from this common reference group in either study. In addition, there were no linear associations between WMC (Study 1) or task engagement (Study 2) with intrusive memories. In light of the absence of condition effects and associations between the critical concepts, we believe there to be little reason to expect an interaction effect in a full factorial study.

However, following this valuable comment, we adapted parts of the manuscript accordingly. Specifically, we have revised our discussion of Study 2 on p.28, clarifying that Study 2 indeed addressed the combination of high load on WM resources and complex visuospatial stimuli. In addition, we have now extended the discussion of relevant null findings in the literature (also see Reviewer 1, point 3). Based on the review comments, we have also extended the discussion of avenues for future research that we believe to be the most promising to unravel when, why, and how visuospatial tasks can interfere with intrusive memories (pp.27-31).
